# Prevalence and correlates of addictive eating behaviours in a large cohort of Australian adolescents

**DOI:** 10.1177/00048674231165201

**Published:** 2023-04-10

**Authors:** Scarlett Smout, Lauren A Gardner, Katrina E Champion, Bridie Osman, Ivana Kihas, Louise Thornton, Maree Teesson, Nicola C Newton, Tracy Burrows

**Affiliations:** 1 The Matilda Centre for Research in Mental Health and Substance Use, The University of Sydney, Darlington, NSW, Australia; 2 School of Medicine and Public Health, The University of Newcastle, Callaghan, NSW, Australia; 3 School of Health Sciences, College of Health, Medicine and Wellbeing, The University of Newcastle, Callaghan, NSW, Australia

**Keywords:** Psychology, nutrition, adolescence, youth, paediatric, disordered eating, food addiction, alcohol, tobacco, energy drink, sugar-sweetened beverage

## Abstract

**Objective:**

Research shows highly palatable foods can elicit addictive eating behaviours or ‘food addiction’. Early adolescence is theorised to be a vulnerable period for the onset of addictive eating behaviours, yet minimal research has examined this. This study explored the prevalence and correlates of addictive eating behaviours in a large early adolescent sample.

**Methods:**

6640 Australian adolescents (M_age_ = 12.7 ± 0.5, 49%F) completed an online survey. Addictive eating was measured with the Child Yale Food Addiction Scale (YFAS-C). Negative-binomial generalised linear models examined associations between addictive eating symptoms and high psychological distress, energy drink consumption, sugar-sweetened beverage (SSB) consumption, alcohol use, and cigarette use.

**Results:**

Mean YFAS-C symptom criteria count was 1.36 ± 1.47 (of 7). 18.3% of participants met 3+ symptoms, 7.5% endorsed impairment and 5.3% met the diagnostic threshold for food addiction. All examined behavioural and mental health variables were significantly associated with addictive eating symptoms. Effects were largest for high psychological distress and cigarette use; with those exhibiting high psychological distress meeting 0.65 more criteria (95%CI = 0.58–0.72, *p* < 0.001) and those who smoked a cigarette meeting 0.51 more criteria (95%CI = 0.26–0.76, *p* < 0.001). High psychological distress and consumption of SSB and energy drinks remained significant when modelling all predictors together.

**Conclusion:**

In this large adolescent study, addictive eating symptoms were common. Further research should establish directionality and causal mechanisms behind the association between mental ill-health, alcohol and tobacco use, and addictive eating behaviours. Cross-disciplinary prevention initiatives that address shared underlying risk factors for addictive eating and mental ill-health may offer efficient yet substantial public health benefits.

## Introduction

Research suggests that highly palatable foods (processed foods high in sugar, salt, and fat) can have addictive properties and, for some, can elicit addictive eating behaviours, also known as ‘food addiction’ (Schiestl et al., 2021; Sylvetsky et al., 2020). The Diagnostic and Statistical Manual (DSM) criteria for substance use disorder have been adapted to capture food addiction, which is characterised by addictive-like consumption patterns of highly palatable foods, despite negative health and psychosocial consequences (Davis and Loxton, 2014; Gearhardt et al., 2013). While not yet recognised as a disorder in the DSM, food addiction has some symptom overlap and comorbidity with recognised eating disorders, including binge eating disorder and anorexia nervosa (bulimic-purgative subtype) (Gearhardt et al., 2014; Sanchez et al., 2022). Despite this overlap, food addiction is present in individuals who do not meet the criteria for other eating disorders (Gearhardt et al., 2014), has demonstrated substantial clinical utility (Cassin and Sockalingam, 2021), has unique treatment requirements (Leary et al., 2021), and has been identified as a key barrier to the effectiveness of nutrition and weight loss interventions (Cassin and Sockalingam, 2021; Schiestl et al., 2021). Food addiction is also associated with mental ill-health and reduced quality of life and individuals with lived experience describe a ‘cycle’ of distress to addictive eating to further distress (Lacroix and von Ranson, 2020; Skinner et al., 2021; Van Ostrand, 2015).

Early adolescence (10–15 years) is a vulnerable period for the onset of mental disorders and disordered eating (Solmi et al., 2022), yet there are no large studies on the prevalence and correlates of food addiction in this cohort. Compared with adults, adolescents have greater reward sensitivity and impulsivity, which are the key risk factors for addictive eating behaviours and other substance use (Maxwell et al., 2020; Zhao et al., 2018). There is evidence that the prevalence and severity of food addiction increases with age (Skinner et al., 2021). Thus, prevention and early intervention among adolescents is crucial to reduce the disease burden from food addiction in adulthood. Elucidating the associations between addictive eating behaviours, consumption of other substances and mental ill-health is essential to identify opportunities for cross-disciplinary prevention initiatives targeting nutrition, mental health and/or substance use.

A recent meta-analysis estimated the prevalence of food addiction in adolescent community samples to be 12% (95% CI = 8%–17%), representing a substantial portion of the adolescent population (Yekaninejad et al., 2021). However, this meta-analysis and another recent systematic review (Skinner et al., 2021) have highlighted several key gaps in the literature. First, most studies have been carried out in European countries and many only report the binary presence or absence of food addiction at the ‘diagnostic’ threshold, despite evidence that examining addictive eating symptomology is more appropriate among adolescents (Gearhardt et al., 2013). Few studies investigated the associations between food addiction and use of other substances, and none have explored the relationship between addictive eating behaviours and energy drink consumption. In addition, the studies generally included small sample sizes and spanned a wide age range, thus not allowing the assessment of prevalence and correlates among *early* adolescents. Finally, examination of sociodemographic correlates of addictive eating among adolescents has typically been limited to gender and age in most existing studies, despite a 2020 narrative review highlighting a gap for examination of cultural and linguistic diversity and its association with addictive eating (Lawson et al., 2020).

To fill these gaps, the present study aimed to:

1. Establish the prevalence and sociodemographic correlates of addictive eating behaviours in a large general population sample of Australian early adolescents aged 11–14 years
2. Examine associations with high psychological distress, alcohol and tobacco use, energy drink consumption, and sugar-sweetened beverage (SSB) consumption.

## Methods

### Participants and procedure

This study utilises baseline data collected from July to November 2019 as part of a cluster randomised controlled trial of ‘Health4Life’; an eHealth intervention targeting multiple lifestyle risk behaviours (Teesson et al., 2020). A total of 71 secondary schools across Australia (37 schools from New South Wales [NSW], 18 from Queensland [QLD], and 16 from Western Australia [WA]) were recruited through convenience sampling. This included schools from the independent (*n* = 38), government (*n* = 24) and Catholic (*n* = 9) sectors. All year 7 students were eligible to participate; however, student and parental consent were required. From a possible total of 9280 students across the 71 schools, 7164 (77.2%) received parental consent, and of these, 6801 (94.9%) provided personal consent. Eligible students completed an online self-report questionnaire in a supervised classroom setting, and were instructed to ask their teacher to explain any questions that they did not understand. Ethical approval was provided by the University of Sydney Human Research Ethics Committee (2018/882), NSW Department of Education (SERAP No. 2019006), the University of QLD (2019000037), Curtin University (HRE2019-0083) and relevant Catholic school committees. Further details, including sample size calculations, recruitment procedures and consent procedures, are in the published study protocol (Teesson et al., 2020).

### Measures

#### Demographic characteristics

Demographic variables included age, gender identity, school geographic remoteness (major city or regional), cultural and linguistic diversity (CALD), and relative family affluence. School geographic remoteness (major city or regional) was used as a proxy for participant geographic remoteness, as many participants did not know their postcode. CALD was defined as participants who were born in a non-English speaking country and/or primarily speak a language other than English at home, per recommendations from a recent Australian review (Pham et al., 2021). As people from CALD backgrounds are more likely to experience systemic barriers to healthcare and poorer health literacy (Henderson and Kendall, 2011), this was deemed an important covariate for the present study. Family affluence was measured using the Family Affluence Scale III (FASIII), which has demonstrated good test–retest reliability (*r* = 0.9) and strong correlation with parental report (Torsheim et al., 2016). The FASIII generates an individual score based on a set of indicators of wealth, which is then transformed to generate a ridit score that indicates the participants’ relative family affluence within the overall sample (Torsheim et al., 2016).

#### Addictive eating

Addictive eating behaviours were measured using the Child Yale Food Addiction Scale (YFAS-C), adapts DSM substance use disorder criteria to consumption of highly palatable foods (Gearhardt et al., 2013). Respondents received examples of these highly palatable foods (“sweets, carbs, salty snacks, fatty foods and sugary drinks”). They then endorsed frequency over the past 12 months (never, rarely, sometimes, very often, always) against 18 statements regarding their consumption of these foods, including ‘I eat until my stomach hurts or I feel sick’ and ‘I need to eat more to get the good feelings I want (eg. happy, calm)’. Respondents then provided binary responses (yes/no) to seven further statements regarding their experiences in the past 12 months, including ‘the way I eat has made me feel sad, nervous, or guilty’ and ‘I am able to cut down on certain foods’. Responses to the 25 total statements were summed against seven symptom criteria modelled off the DSM criteria – (1) loss of control, (2) inability to cut down, (3) substantial time spent to obtain/use/recover, (4) giving up important social/occupational/recreational activities, (5) continued use despite negative consequences, (6) tolerance, and (7) withdrawal. An eighth criteria – clinically significant impairment – is met if the respondent endorses either or both of the statements ‘the way I eat makes me really unhappy’ and ‘the way I eat causes me problems (e.g. problems at school, with my parents, with my friends)’. Individuals who meet three or more symptom criteria and endorse ‘impairment’ meet the proxy ‘food addiction diagnosis’. However, for younger adolescents in a non-clinical population, Gearhardt et al. (2013) recommend utilisation of the symptom count as a more sensitive outcome metric compared to proxy diagnosis, as evidence shows that impairment occurs in later adolescence and early adulthood but is preceded by elevated symptoms (Gearhardt et al., 2013). As such, symptom count was used as the primary outcome for this study. To minimise missing data, the symptom count was prorated if the participant was missing data for only one of seven of the symptom criteria. The YFAS-C has demonstrated good internal consistency (α = 0.86) and moderate-to-good convergent validity with similar measures (*r* = 0.46–0.61, *p* = 0.01) (Gearhardt et al., 2013).

#### Alcohol and tobacco use

Alcohol use was assessed using the item: ‘Have you had a full standard alcoholic drink in the past 6 months?’ (yes/no). A pictorial chart was supplied to assist participants in identifying what constitutes an Australian standard drink. Tobacco use was captured using the item ‘In the past 6 months, have you tried cigarette smoking, even one or two puffs?’ (yes/no), adapted from the Standard High School Youth Risk Behaviour Survey (Centers for Disease Control and Prevention, 2019). These low use thresholds were selected as prevalence of cigarette and alcohol use is low among early adolescents in Australia Guerin and White (2018) and *any* use in this age group is of note as evidence suggests that earlier onset of use predicts greater likelihood of dependence (Huggett et al., 2019; Ohannessian et al., 2015).

#### SSB and energy drink consumption

While the YFAS-C lists sugary drinks as an example in the list of highly palatable foods, the vast majority of existing food addiction research has only examined associations with consumption of *food*. As adolescence is the period in which consumption of SSB peaks (Australian Bureau of Statistics, 2011), this was deemed an important relationship to investigate. Furthermore, the YFAS-C does not explicitly mention energy drink consumption, and no studies to-date have examined associations between food addiction and energy drinks. Using items from the Student Physical Activity and Nutrition Survey (SPANS), students were presented with visual cues on the number of metric cups in a range of beverage bottle/can sizes and asked to report how many cups of SSB (soft drink, cordials, or sports drinks) they ‘usually’ consume (Hardy et al., 2016). Response options ranged from ‘Never/Rarely drink’ to ‘2 or more cups a day’. Energy drink consumption was also measured using items from SPANS (Hardy et al., 2016). Students were given examples (e.g. ‘Mother, V, Red Bull’) and separately reported their usual consumption of small sized energy drinks (<500 mL) and large sized energy drinks (⩾500 mL), again with options from ‘Never/Rarely drink’ to ‘2 or more cups a day’. The small and large serving size items were combined into a four-level categorical variable: ‘Never/rarely drink’, ‘low consumption’ (1 small OR 1 large serve or less per week), ‘moderate consumption’ (2–4 small serves per week OR 1 small serve per week AND 1 large serve per week), and ‘high consumption’ (all higher options). The SPANS questionnaire has no available psychometric evaluation but was selected to facilitate comparability with data from the three waves of SPANS, which have collectively encompassed over 20,000 Australian children and adolescents (Hardy et al., 2016).

#### Mental health

The Kessler six-item scale (K6) was used to capture psychological distress and has demonstrated good internal consistency (α = 0.84) and predictive validity in adolescents (Mewton et al., 2016). Participants report frequency of feelings such as nervousness, hopelessness, restlessness and worthlessness. A summed psychological distress symptom score (0–24) is dichotomised, with scores ⩾13 indicating high psychological distress, at levels tantamount to a probable mental, behavioural or emotional disorder (Green et al., 2010; Peiper et al., 2015).

### Statistical analysis

Descriptive analysis was used to examine YFAS-C symptom endorsement and mean symptom count by demographic factors (gender, age, relative family affluence, geographic location and CALD status); mental health status (presence or absence of high psychological distress); and behavioural factors (alcohol use, cigarette use and energy drink consumption). Next, generalised linear models, with cluster-robust standard errors to account for school-level clustering, were used to examine the relationship between YFAS-C symptom count, high psychological distress, energy drink consumption, SSB consumption, alcohol use and smoking. The models adjusted for gender, age, relative family affluence, geographic location and CALD status. For symptom score, the possible range was low (0–7) and had a zero inflated distribution (Supplementary Figure 1). As such, models with a Poisson and negative binomial distribution were tested and a negative binomial distribution was selected due to superior model fit (according to Akaike information criterion [AIC] and Bayesian information criterion [BIC] values). To examine characteristics associated with higher odds of meeting three or more symptom criteria, generalised linear models with a binomial distribution and cluster-robust standard errors. Checks for multicollinearity were performed and no collinearity was found (variable inflation factor all < 1.3 in model with all covariates included). Due to the placement of the YFAS-C towards the end of the survey and students having only one school period to complete the survey, there was a substantial amount of missing data for the YFAS-C (*n* = 1087 [16% of participating students] missing). This missingness was interrogated to establish any significant differences by demographic factors, behavioural factors and high psychological distress. All analyses were conducted in R Studio version 2022.2.0.443.

## Results

### Participant characteristics

The final baseline Health4Life data set included 6640 students (M_age_ = 12.7; SD = 0.5; 50.6% Male-identifying, 48.9% Female-identifying, 0.5% non-binary) (Snijder et al., 2021). Participants were from NSW (53.2%), QLD (26.9%) and WA (19.8%) and the majority (89.1%) were attending schools in major city areas (remaining 10.9% in regional areas). 12.2% of participants were culturally and linguistically diverse. 11% of students (*n* = 705) consumed five or more cups of SSB in a usual week; 23.8% consumed energy drink/s (any volume) in a usual week (*n* = 1540); 1.5% smoked a cigarette in the prior 6 months (*n* = 97); and 2.9% consumed one or more standard alcoholic drinks in the prior 6 months (*n* = 181). High psychological distress at a level indicating a probable mental, behavioural or emotional disorder (K6 **⩾** 13) was present in 14.1% of students (*n* = 886).

### Addictive eating symptoms and missing data

**Figure 1.**
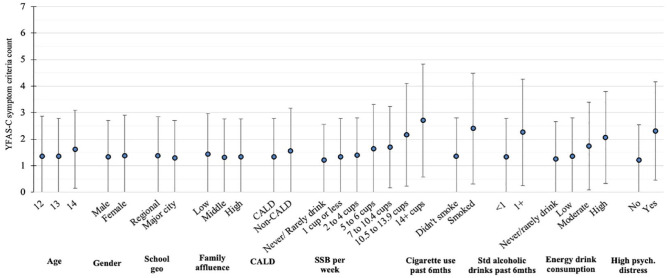
Mean YFAS-C symptom criteria count (with SD bars), by participant characteristic.

**Figure 2.**
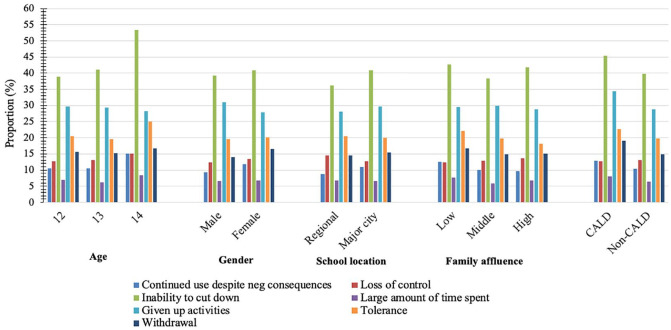
Proportion of students meeting each YFAS-C symptom criterion by sociodemographic group.

As discussed in the methods, due to the placement of the YFAS-C towards the end of the survey and students having only one school period to complete the survey, there was a substantial amount of missing data for the YFAS-C (*n* = 1087 [16% of participating students] missing). Those who completed the YFAS-C (*n* = 5553) met a mean of 1.36 symptom criteria (SD = 1.46), of a possible seven YFAS-C criteria. Mean symptom count by participant characteristic is summarised in Figure 1 and Table 1. The most frequently met symptoms were persistent desire or repeated unsuccessful attempts to cut down (‘inability to cut down’, *n* = 2296 [40.4%]), important social, occupational or recreational activities given up or reduced (‘activities given up’, *n* = 1671 [29.5%]), and ‘tolerance’ (*n* = 1126 [20.0%]). The prevalence of each symptom criteria by sociodemographic group is summarised in Figure 2 and Supplementary Table 1. Three or more symptom criteria (*n* = 1022) were met by 18.2% of participants, 7.5% (*n* = 421) met clinically significant impairment and 5.3% (*n* = 292) met the YFAS-C ‘diagnosis’ of food addiction (requiring 3+ symptoms *and* clinical impairment).

**Table 1.** Participant characteristics and mean YFAS-C symptom count.

		Sample portion, *N* (%)	Mean YFAS-C symptom count (SD) among portion of sample with completed YFAS-C (*n* = 5553)
Age	11	9 (0.1%)	Redacted due to low number of participants
	12	2365 (35.8%)	1.36 (1.50)
	13	4158 (63%)	1.35 (1.44)
	14	71 (1.1%)	1.62 (1.47)
Gender identity	Male	3311 (50.6%)	1.33 (1.38)
	Female	3204 (49%)	1.38 (1.53)
	Non-binary/gender fluid	30 (0.5%)	Redacted
School geographic remoteness	Regional	727 (10.9%)	1.37 (1.47)
	Major city	5913 (89.1%)	1.30 (1.41)
Relative family affluence (ridit adjusted)	Low	1752 (29.1%)	1.44 (1.52)
	Middle	2667 (44.3%)	1.32 (1.44)
	High	1596 (26.5%)	1.34 (1.42)
Culturally and Linguistically Diverse (CALD)	Yes	808 (12.2%)	1.34 (1.44)
	No	5810 (87.8%)	1.56 (1.60)
Sugar-sweetened beverage consumption	Never/Rarely drink	2561 (39.6%)	1.21 (1.35)
	1 cup or less per week	2142 (33.1%)	1.34 (1.44)
	2–4 cups per week	1058 (16.4%)	1.39 (1.41)
	5–6 cups per week	275 (4.3%)	1.64 (1.67)
	7–10.4 cups per week	186 (2.9%)	1.70 (1.53)
	10.5–13.9 cups per week	98 (1.5%)	2.17 (1.94)
	14+ cups per week	146 (2.3%)	2.71 (2.13)
Cigarette smoking in prior 6 months	Didn’t smoke	6209 (98.5%)	1.35 (1.45)
	Smoked	97 (1.5%)	2.40 (2.09)
Consumption of one or more full standard alcoholic drinks in prior 6 months	No	6165 (97.1%)	1.34 (1.44)
	Yes	181 (2.9%)	2.26 (2.01)
Energy drink consumption	Never/rarely drink	4910 (76.1%)	1.26 (1.40)
	Low consumption	660 (10.2%)	1.36 (1.44)
	Moderate consumption	374 (5.8%)	1.74 (1.66)
	High consumption	506 (7.8%)	2.06 (1.73)
High psychological distress	No	5392 (85.9%)	1.21 (1.33)
	Yes	886 (14.1%)	2.31 (1.85)
Total sample: Addictive eating symptom count			1.36 (1.46)

YFAS-C: Child Yale Food Addiction Scale; SD: standard deviation.

Supplementary Table 2 summarises the difference between participants that completed the YFAS-C and those with missing YFAS-C data. There were no significant differences for any characteristics aside from age and gender, with a higher portion of 12-year-olds and a higher portion of males having missing data than 13-year-olds and females, respectively. As age and gender were not significantly associated with YFAS-C symptom criteria count (see ‘Addictive eating correlates’), this is unlikely to have resulted in non-response bias.

### Addictive eating correlates

As shown in Figure 2, there were minimal differences in unadjusted symptom endorsement between sociodemographic groups, aside from higher mean symptoms in CALD participants compared to non-CALD participants. This was supported by negative binomial GLMs which demonstrated that CALD participants had significantly higher YFAS-C symptom count than non-CALD participants (Estimated = 0.16 [95% CI = 0.08–0.23], *p* < 0.001). There were no significant differences by age, gender, affluence or geographic remoteness (see Supplementary Table 3).

As shown in Figure 1 and Table 1, the raw mean symptom count was highest in those who: consumed 14+ cups of SSB per week (2.71 [2.13]), consumed high amounts of energy drinks (2.06 [1.73]), had smoked a cigarette in the prior six months (2.40 [2.09]), had consumed one or more standard alcoholic drinks in the prior six months (2.26 [2.01]) or had high psychological distress (2.31 [1.85]). Negative binomial GLMs adjusting for age, gender, CALD status and school clustering demonstrated that – when modelled alone – each predictor was significantly associated with YFAS-C symptom criteria count (see Supplementary Table 4). Effect sizes were largest for high psychological distress and cigarette use; with those exhibiting high psychological distress meeting 0.65 more criteria on average (95% CI = 0.58–0.72, *p* < 0.001) and those who had smoked a cigarette meeting 0.51 more criteria on average (95% CI = 0.26–0.76, p < 0.001).

SSB consumption, energy drink consumption and high psychological distress remained significantly associated with YFAS-C symptom count when modelling all predictors together, as did CALD status (Table 2). On average, those who consumed 14 or more cups of SSB per week met 0.54 more symptom criteria than those who never/rarely drank SSB (95% CI = 0.37–0.70, *p* < 0.001); those who consumed high amounts of energy drinks met 0.26 more criteria than those who never/rarely drank SSB (95% CI = 0.16–0.36, *p* < 0.001); and those with high psychological distress met 0.58 more criteria than those without high psychological distress (95% CI = 0.51–0.65, *p* < 0.001).

**Table 2.** Negative-binomial generalised linear regression model examining associations between predictors and YFAS-C symptom score.

		Estimated (95% CI) symptom difference (out of a possible seven symptoms)	*p*-value
Sugar-sweetened beverage consumption	Never/Rarely drink	Reference	<0.001
	1 cup or less per week	0.10 (0.03–0.16)	
	2–4 cups per week	0.11 (0.01–0.21)	
	5–6 cups per week	0.15 (0.00–0.30)	
	7–10.4 cups per week	0.29 (0.11–0.46)	
	10.5–13.9 cups per week	0.42 (0.23–0.61)	
	14+ cups per week	0.54 (0.37–0.70)	
Cigarette smoking in prior 6 months	Didn’t smoke	Reference	0.11
	Smoked	0.19 (–0.04–0.42)	
Consumption of one or more full standard alcoholic drinks in prior 6 months	No	Reference	0.13
	Yes	0.11 (–0.03–0.26)	
Energy drink consumption	Never/rarely drink	Reference	<0.001
	Low consumption	–0.01 (–0.10–0.08)	
	Moderate consumption	0.20 (0.11–0.29)	
	High consumption	0.26 (0.16–0.36)	
High psychological distress	No	Reference	<0.001
	Yes	0.58 (0.51–0.65)	
Age^a^	12	Reference	0.996
	13	0.00 (–0.07–0.07)	
Gender identity^a^	Male	Reference	0.044
	Female	0.07 (0.00–0.14)	
Culturally and Linguistically Diverse (CALD)	Not CALD	Reference	0.006
	CALD	0.10 (0.03–0.16)	
Relative family affluence (ridit-adjusted)	Low	0.05 (–0.01–0.11)	0.327
	Middle	Reference	
	High	0.01 (–0.05–0.08)	

YFAS-C: Child Yale Food Addiction Scale; CI: confidence interval.

a Participants aged 11 or 14 and participants identifying as non-binary gender were excluded from regressions due to low numbers.

Table 3 summarises the results of a model examining the associations of all predictors with odds of meeting three or more YFAS-C symptom criteria. After adjusting for other predictors, those who consumed 14 or more cups of SSB per week were 225% more likely to meet 3+ symptoms than those who never/rarely drank SSB (OR = 3.25, 95% CI = 2.05–5.17, *p* < 0.001); those who consumed high amounts of energy drinks were 82% more likely to meet 3+ symptoms than those who never/rarely drank energy drinks (OR = 1.82, 95% CI = 1.40–2.38, *p* < 0.001); and those with high psychological distress were 205% more likely to meet 3+ symptoms than those without high psychological distress (OR = 3.05, 95% CI = 2.56–3.63, *p* < 0.001). CALD background and female gender identity were associated with 26% and 18% higher likelihood of meeting 3+ symptom criteria, respectively (CALD OR = 1.26, 95% CI = 1.03–1.54, *p* = 0.02; gender OR = 1.18, 95% CI = 1.00–1.39, *p* = 0.04).

**Table 3.** Associations between predictors and odds of meeting three or more YFAS-C symptom criteria.

		AOR (95% CI)	*p*-value
Sugar-sweetened beverage consumption	Never/Rarely drink	Reference	Reference
	1 cup or less per week	1.19 (1.02–1.38)	0.024
	2–4 cups per week	1.07 (0.85–1.35)	0.547
	5–6 cups per week	1.20 (0.85–1.70)	0.307
	7–10.4 cups per week	1.45 (0.92–2.27)	0.110
	10.5–13.9 cups per wee	2.05 (1.24–3.40)	0.005
	14+ cups per week	3.25 (2.05–5.17)	<0.001
Cigarette smoking in prior 6 months	Didn’t smoke	Reference	0.968
	Smoked	0.99 (0.50–1.93)	
Consumption of one or more full standard alcoholic drinks in prior 6 months	No	Reference	0.016
	Yes	1.59 (1.09–2.33)	
Energy drink consumption	Never/rarely drink	Reference	Reference
	Low consumption	1.03 (0.80–1.31)	0.83
	Moderate consumption	1.66 (1.28–2.16)	<0.001
	High consumption	1.82 (1.40–2.38)	<0.001
High psychological distress	No	Reference	<0.001
	Yes	3.05 (2.56–3.63)	
Age^a^	12	Reference	0.34
	13	0.92 (0.79–1.09)	
Gender identity^a^	Male	Reference	0.04
	Female	1.18 (1.00–1.39)	
Culturally and Linguistically Diverse (CALD) – Yes	Not CALD	Reference	0.02
	CALD	1.26 (1.03–1.54)	
Relative family affluence (ridit adjusted)	Low	1.10 (0.94–1.29)	0.22
	Middle	Reference	Reference
	High	0.87 (0.72–1.05)	0.15

CI: Confidence interval; AOR: Adjusted odds ratio.

a Participants aged 11 or 14 and participants identifying as non-binary gender were not included in regressions due to low numbers.

## Discussion

This study is the first to explore the prevalence and patterns of addictive eating symptoms, and associations with mental ill-health, substance use, SSB and energy drink consumption in adolescents. In this large cohort of Australian adolescents aged 11–14, the mean food addiction symptom count was 1.36 out of 7 (SD = 1.46). Nearly one in five participants (18.2%) met three or more symptom criteria, 7.5% met clinically significant impairment, and one in 20 (5.3%) met the YFAS-C ‘diagnosis’ of food addiction. With evidence that prevalence increases with age (Skinner et al., 2021), this relatively common presentation of addictive eating behaviours in such a young cohort warrants attention.

The mean symptom count and prevalence of food addiction were slightly lower in this sample than in the most recent meta-analysis of community sampled adolescents, which placed mean symptom count at 1.54 and food addiction at 12% (95% CI = 8–17%) (Yekaninejad et al., 2021). However, this is likely due to addictive eating being shown to be higher among older adolescents (Skinner et al., 2021) and the meta-analysis not including any studies conducted solely among early adolescents (Yekaninejad et al., 2021). Consistent with other research (Skinner et al., 2021), the prevalence of meeting the diagnostic threshold of food addiction was higher in females than males (6.7% and 3.6%, respectively), despite similar mean symptom criteria counts (males: 1.33, females: 1.38). The difference in diagnostic level food addiction is driven by gender differences in endorsement of clinically significant distress or impairment, with males less likely to endorse this criterion than females, again consistent with other research (Skinner et al., 2021).

In this study, the most frequently met symptoms were persistent desire or repeated unsuccessful attempts to cut down (40.4%), important social, occupational, or recreational activities given up or reduced (29.5%), and tolerance (20.0%). The high prevalence of repeated attempts to cut down is perhaps unsurprising given that a recent US nationally representative survey found that 50% of 8–15 year-olds had tried to lose weight in the past year, with 83.5% of those doing so by attempting to cut down consumption of sweet/fatty foods. Indeed, systematic reviews among adolescents (Skinner et al., 2021) and adults (Burrows et al., 2018) also identified this to be the most prevalent YFAS symptom.

Cultural and linguistic diversity was found to be associated with a higher addictive eating symptom criteria count. To the authors’ knowledge, this association has not been examined in previous adolescent samples, with a recent narrative review highlighting this gap (Lawson et al., 2020). This should be considered in the design of prevention interventions as they may need to be tailored to meet the needs of CALD adolescents. Age was not significantly associated with addictive eating behaviours in this study, in contrast to other studies, which have indicated that food addiction symptoms increased with age (Skinner et al., 2021). However, this is likely due to the small age range of the present study, with only 12 and 13 year-olds included in regressions due to very small numbers of 11 and 14 year-olds. Gender identity was not associated with addictive eating symptoms (aside from a weak association in the full multi-predictor model), echoing findings from the aforementioned meta-analysis (Yekaninejad et al., 2021). This is of note, given significantly higher prevalence of eating disorders among adolescent females than males, further indicating that addictive eating may be a unique construct despite some overlap with symptoms of eating disorders (Gearhardt et al., 2014).

When examining the correlates of addictive eating symptoms individually, higher symptom counts were strongly associated with alcohol use, cigarette use, higher SSB consumption, higher energy drink consumption, and high psychological distress after adjusting for gender, age and CALD status. In a model that included all predictors, SSB consumption, energy drink consumption and high psychological distress remained significantly associated with addictive eating symptoms. These findings align with previous research among an older Dutch adolescent sample, which showed that alcohol, tobacco and SSB use were associated with addictive eating symptoms (Mies et al., 2017). The finding that high psychological distress was associated with addictive eating echoed the results of a systematic review, which found that that anxiety, depression and psychological distress were associated with food addiction (Skinner et al., 2021). The finding that energy drink consumption was associated with addictive eating behaviours is a novel contribution as no existing research has examined this link in adolescents or adults.

While this study is cross-sectional, thus precluding investigation of directionality and causal mechanisms, several mechanisms may explain findings. There may be a bi-directional relationship between food addiction and mental disorders, with consumption of highly processed foods linked to increased adverse mental health (Lane et al., 2022), and highly processed foods being used as a maladaptive coping mechanism for negative emotions (Schulte et al., 2017). The neurotransmitter dopamine may play a role, as emerging research indicates a compromised dopamine motive system in individuals with food addiction (Campana et al., 2019; Volkow et al., 2017), and highly processed foods, caffeine, alcohol and nicotine all activate or enhance the availability of dopamine when consumed or used (Criscitelli and Avena, 2016; Volkow et al., 2015). In addition, reduced levels of the hormone oxytocin have been identified in individuals with mental disorders and substance use disorders, and emerging evidence suggests that oxytocin may be implicated in food addiction; however, a recent review found inconclusive evidence (Skinner et al., 2018). Finally, personality traits may play a role. A study among a community sample of adults found that individuals scoring higher on anxiety sensitivity and impulsivity personality traits were more likely to meet food addiction criteria (Burrows et al., 2017), and impulsivity is associated with early onset adolescent alcohol and tobacco use (Krank et al., 2011; Newton et al., 2016).

Food addiction has been identified as a key barrier to the effectiveness of nutrition and weight loss interventions (Cassin and Sockalingam, 2021; Schiestl et al., 2021), and is often comorbid with DSM-recognised eating disorders (Gearhardt et al., 2014; Sanchez et al., 2022), which are highly prevalent among Australian adolescents (Mitchison et al., 2020). As such, food addiction should be considered in the design, implementation and evaluation of nutrition interventions. In addition, associations found between addictive eating behaviours and alcohol use, cigarette use, high psychological distress, and energy drink consumption highlight an opportunity for cross-disciplinary prevention initiatives that address shared risk and protective factors.

### Strengths

The present study represents the largest sample to-date to assess addictive eating behaviours among adolescents, with the second largest including 2653 Dutch young people (aged 14–21) (Mies et al., 2017). Moreover, this study fills a gap for the investigation of addictive eating among *early* adolescents, which represents a unique developmental period. In addition, the majority of existing studies among adolescents report on presence of clinically significant food addiction only, despite evidence that symptom count should be used in adolescent community samples (Gearhardt et al., 2013). As such, this study provides a novel contribution to current research through the presentation of prevalence and distribution of symptom count in addition to diagnosis.

### Limitations and opportunities for future research

Although large, the included sample is not nationally representative so findings cannot be generalised to the population. Future nationally representative studies among adolescents should consider measuring addictive eating to glean prevalence in the general population. All measures used in this study were self-report so despite strong psychometric properties, they may be prone to reporting bias. Since the time of data collection, an updated version of the YFAS-C has been released – the YFAS-C 2.0 (Schiestl and Gearhardt, 2018). While strong concordance between the YFAS and the YFAS 2.0 has been demonstrated, findings should be interpreted with this limitation in mind. In addition, due to the placement of the YFAS-C towards the end of the survey and students having only one school period to complete the survey, there was a substantial amount of missing data for the YFAS-C. Missing data analysis demonstrated that this is unlikely to have resulted in non-response bias; however this remains a limitation. The online questionnaire was in English, and – although a research assistant administered the survey for vision impaired students – it is possible that lower literacy students or linguistically diverse students may have misinterpreted questions or left questions unanswered. To minimise this, students were instructed to ask their teacher to explain any questions that they didn’t understand. In addition, missing data analysis showed that CALD students did not have significantly more missing data for the YFAS-C.

This study fills a gap in the literature by examining the associations between addictive eating symptoms and beverage consumption (SSB and energy drinks), however future research should examine associations with *both* beverage and food consumption in early adolescents as sugary drink consumption often occurs in tandem with highly palatable food. E-cigarette use (or ‘vaping’) was not measured so analysis of tobacco use was restricted to combustible cigarette use. Given recent increases in vaping among adolescents, (Guerin and White, 2018) future research should examine the relationship between vaping and addictive eating to assess whether the association found with combustible cigarette use remains. The cross-sectional design of this study does not allow for assessment of directional associations. Future research should examine the relationship between substance use, mental ill-health and addictive eating behaviours longitudinally, to identify directionality. Finally, further research should be conducted to elucidate shared, modifiable risk mechanisms across addictive eating, alcohol and tobacco use, energy drink consumption, and SSB consumption to inform cross-disciplinary prevention and early intervention.

## Supplemental Material

sj-docx-1-anp-10.1177_00048674231165201 – Supplemental material for Prevalence and correlates of addictive eating behaviours in a large cohort of Australian adolescents Click here for additional data file. Supplemental material, sj-docx-1-anp-10.1177_00048674231165201 for Prevalence and correlates of addictive eating behaviours in a large cohort of Australian adolescents by Scarlett Smout, Lauren A Gardner, Katrina E Champion, Bridie Osman, Ivana Kihas, Louise Thornton, Maree Teesson, Nicola C Newton and Tracy Burrows in Australian & New Zealand Journal of Psychiatry
