# Supplementary material for: Prevalence and correlates of addictive eating behaviours in a large cohort of Australian adolescents
Source: Aust N Z J Psychiatry. 2023 Apr 10;57(8):1172–83. doi: 10.1177/00048674231165201 (PMC10363940; doi:10.1177/00048674231165201)
Supplement: sj-docx-1-anp-10.1177_00048674231165201 – Supplemental material for Prevalence and correlates of addictive eating behaviours in a large cohort of Australian adolescents [file sj-docx-1-anp-10.1177_00048674231165201.docx]

## Supplementary Materials:

**Supplementary Figure 1. Distribution of YFAS-C symptom count**


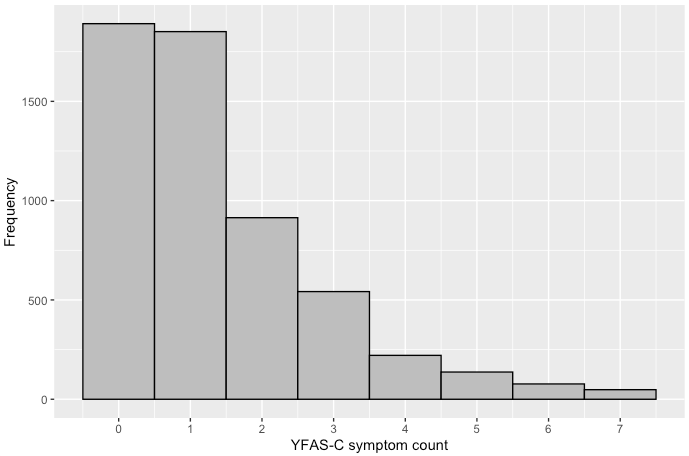


Supplementary Table 1. YFAS-C symptom criteria endorsement by participant characteristics

|  |  | Continued use despite negative consequences | Loss of control | Inability to cut down | Large amount of time spent | Given up activities | Tolerance | Withdrawal | Clinical significance | Food addiction "diagnosis" |
| --- | --- | --- | --- | --- | --- | --- | --- | --- | --- | --- |
| Age | 11 | Redacted due to low number of participants risking re-identification of data | | | | | | | | |
|  | 12 | 206 (10.6%%) | 251 (12.7%) | 766 (38.8%) | 138 (7.0%) | 586 (29.7%) | 398 (20.4%) | 306 (15.6%) | 158 (8.1%) | 114 (5.9%) |
|  | 13 | 376 (10.6%) | 472 (13.1%) | 1482 (41.0%) | 227 (6.3%) | 1055 (29.3%) | 702 (19.6%) | 548 (15.2%) | 255 (7.1%) | 172 (4.9%) |
|  | 14 | 9 (15.0%) | 9 (15.0%) | 32 (53.3%) | 5 (8.3%) | 17 (28.3%) | 15 (25.0%) | 10 (16.7%) | 2 (3.3%) | 1 (1.7%) |
| Gender identity | Male | 253 (9.3%) | 342 (12.3%) | 1092 (39.3%) | 182 (6.6%) | 856 (30.9%) | 539 (19.6%) | 386 (14.0%) | 141 (5.1%) | 97 (3.6%) |
|  | Female | 324 (11.8%) | 377 (13.5%) | 1146 (40.9%) | 186 (6.7%) | 778 (27.8%) | 559 (20.2%) | 462 (16.5%) | 263 (9.4%) | 183 (6.7%) |
|  | Non-binary | Redacted due to low number of participants risking re-identification of data | | | | | | | | |
| School geographic remoteness | Regional | 52 (8.8%) | 87 (14.5%) | 216 (36.2%) | 40 (6.7%) | 167 (28.1%) | 122 (20.5%) | 87 (14.6%) | 48 (8.1%) | 28 (4.8%) |
|  | Major city | 543 (10.9%) | 650 (12.8%) | 2080 (40.9%) | 336 (6.6%) | 1504 (29.7%) | 1004 (20.0%) | 784 (15.5%) | 373 (7.4%) | 264 (5.3%) |
| Relative family affluence (ridit adjusted) | Low | 203 (12.6%) | 203 (12.4%) | 697 (42.6%) | 125 (7.6%) | 482 (29.5%) | 359 (22.1%) | 273 (16.7%) | 144 (8.8%) | 101 (6.3%) |
|  | Middle | 240 (10.0%) | 316 (12.9%) | 940 (38.3%) | 142 (5.8%) | 730 (29.8%) | 479 (19.7%) | 361 (14.8%) | 180 (7.4%) | 118 (4.9%) |
|  | High | 139 (9.6%) | 202 (13.7%) | 617 (41.7%) | 100 (6.8%) | 423 (28.8%) | 265 (18.1%) | 220 (15.0%) | 87 (5.9%) | 65 (4.5%) |
| Culturally & linguistically diverse (CALD) | Yes | 88 (12.9%) | 88 (12.7%) | 315 (45.4%) | 56 (8.1%) | 236 (34.3%) | 155 (22.6%) | 131 (19.1%) | 59 (8.6%) | 44 (6.5%) |
|  | No | 506 (10.4%) | 648 (13.0%) | 1978 (39.8%) | 319 (6.4%) | 1431 (28.8%) | 968 (19.7%) | 738 (14.9%) | 361 (7.3%) | 247 (5.1%) |
| Sugar-sweetened beverage consumption | Never/ Rarely drink | 206 (9.3%) | 245 (10.9%) | 863 (38.2%) | 93 (4.1%) | 610 (27.1%) | 369 (16.5%) | 312 (13.9%) | 157 (7.0%) | 104 (4.7%) |
|  | 1 cup or less per week | 184 (10.0%) | 247 (13.2%) | 736 (39.4%) | 119 (6.4%) | 555 (29.8%) | 359 (19.4%) | 290 (15.6%) | 152 (8.2%) | 101 (5.5%) |
|  | 2 to 4 cups per week | 98 (10.6%) | 116 (12.4%) | 385 (41.1%) | 66 (7.1%) | 286 (30.7%) | 214 (23.0%) | 135 (14.5%) | 56 (6.0%) | 38 (4.1%) |
|  | 5 to 6 cups per week | 34 (14.8%) | 40 (17.5%) | 100 (43.5%) | 30 (13.1%) | 76 (33.2%) | 53 (23.0%) | 44 (19.2%) | 14 (6.1%) | 12 (5.3%) |
|  | 7 to 10.4 cups per week | 24 (15.2%) | 26 (15.9%) | 80 (48.8%) | 19 (11.6%) | 44 (26.8%) | 50 (31.1%) | 31 (18.9%) | 10 (6.1%) | 9 (5.7%) |
|  | 10.5 to 13.9 cups per wee | 21 (24.1%) | 21 (23.6%) | 45 (50.0%) | 16 (18.0%) | 37 (41.6%) | 27 (30.7%) | 22 (24.7%) | 13 (14.6%) | 12 (14.0%) |
|  | 14+ cups per week | 28 (23.3%) | 42 (32.8%) | 86 (67.2%) | 33 (25.8%) | 63 (49.2%) | 54 (42.9%) | 37 (29.1%) | 19 (15.0%) | 16 (13.3%) |
| Cigarette use in prior 6mths | Didn't smoke | 573 (10.5%) | 717 (12.9%) | 2240 (40.2%) | 347 (6.2%) | 1632 (29.4%) | 1092 (19.8%) | 834 (15.0%) | 399 (7.2%) | 272 (5.0%) |
|  | Smoked | 21 (22.8%) | 19 (20.4%) | 53 (56.4%) | 28 (29.8%) | 37 (39.4%) | 32 (34.4%) | 34 (36.2%) | 21 (22.3%) | 19 (20.9%) |
| Alcohol use (≥  1 standard drink) in prior 6mths | No | 561 (10.4%) | 698 (12.7%) | 2208 (40.1%) | 338 (6.1%) | 1595 (29.0%) | 1079 (19.8%) | 829 (15.1%) | 390 (7.1%) | 263 (4.9%) |
|  | Yes | 34 (21.7%) | 39 (24.4%) | 85 (53.1%) | 38 (23.8%) | 76 (47.5%) | 47 (29.4%) | 42 (26.3%) | 31 (19.4%) | 29 (18.5%) |
| Energy drink consumption | Never/rarely drink | 412 (9.7%) | 509 (11.8%) | 1687 (39.0%) | 233 (5.4%) | 1207 (28.0%) | 756 (17.7%) | 616 (14.3%) | 312 (7.3%) | 203 (4.8%) |
|  | Low | 57 (9.9%) | 73 (12.6%) | 233 (40.0%) | 45 (7.8%) | 144 (24.9%) | 133 (23.0%) | 97 (16.8%) | 36 (6.2%) | 25 (4.4%) |
|  | Moderate | 48 (15.3%) | 60 (19.0%) | 143 (45.1%) | 26 (8.2%) | 120 (37.9%) | 89 (28.3%) | 61 (19.3%) | 23 (7.3%) | 20 (6.4%) |
|  | High | 77 (17.8%) | 95 (21.2%) | 230 (51.2%) | 72 (16.1%) | 198 (44.2%) | 146 (33.0%) | 95 (21.3%) | 50 (11.2%) | 44 (10.2%) |
| Probable serious mental illness | No | 383 (8.0%) | 562 (11.5%) | 1845 (37.8%) | 252 (5.2%) | 1342 (27.6%) | 832 (17.2%) | 632 (13.0%) | 244 (5.0%) | 161 (3.4%) |
|  | Yes | 212 (27.2%) | 175 (22.1%) | 449 (56.6%) | 124 (15.7%) | 329 (41.6%) | 294 (37.3%) | 239 (30.2%) | 177 (22.4%) | 131 (16.8%) |
| Total sample | | 595 (10.7%) | 737 (13.0%) | 2296 (40.4%) | 376 (6.6%) | 1671 (29.5%) | 1126 (20.0%) | 871 (15.4%) | 421 (7.5%) | 292 (5.3%) |

**Supplementary Table 2. Missing YFAS-C Data**

|  |  | YFAS-C Missing | YFAS-C Captured | Significance of difference |
| --- | --- | --- | --- | --- |
|  | n | 1087 | 5553 |  |
| Age | 12 | 435 (18.4%) | 1930 (81.6%) | <0.001 |
|  | 13 | 623 (15.0%) | 3535 (85.0%) |  |
| Gender | Male | 593 (17.9%) | 2718 (82.1%) | <0.001 |
|  | Female | 461 (14.4%) | 2743 (85.6%) |  |
| School geographic remoteness | Regional | 139 (19.1%) | 588 (80.9%) | 0.038 |
|  | Major city | 895 (15.1%) | 5018 (84.9%) |  |
| Relative family affluence (ridit adjusted) | Low | 140 (8.0%) | 1612 (92.0%) | 0.039 |
|  | Middle | 274 (10.3%) | 2393 (89.7%) |  |
|  | High | 151 (9.5%) | 1445 (90.5%) |  |
| Culturally and linguistically diverse (CALD) | No | 941 (16.2%) | 4869 (83.8%) | 0.96 |
|  | Yes | 132 (16.3%) | 676 (83.7%) |  |
| Sugar-sweetened beverage consumption | Never/ Rarely drink | 354 (13.8%) | 2207 (86.2%) | 0.463 |
|  | 1 cup or less per week | 310 (14.5%) | 1832 (85.5%) |  |
|  | 2 to 4 cups per week | 137 (12.9%) | 921 (87.1%) |  |
|  | 5 to 6 cups per week | 47 (17.1%) | 228 (82.9%) |  |
|  | 7 to 10.4 cups per week | 28 (15.1%) | 158 (84.9%) |  |
|  | 10.5 to 13.9 cups per week | 12 (12.2%) | 86 (87.8%) |  |
|  | 14+ cups per week | 26 (17.8%) | 120 (82.2%) |  |
| Cigarette smoking in prior 6 months | No | 757 (12.2%) | 5452 (87.8%) | 0.1 |
|  | Yes | 6 (6.2%) | 91 (93.8%) |  |
| Consumption of one or more full standard alcoholic drinks in prior 6 months | No | 774 (12.6%) | 5391 (87.4%) | 0.866 |
|  | Yes | 24 (13.3%) | 157 (86.7%) |  |
| Energy drink consumption | Never/rarely drink | 676 (13.8%) | 4234 (86.2%) | 0.549 |
|  | Low consumption (1 small or big serve or less per week) | 90 (13.6%) | 570 (86.4%) |  |
|  | Moderate consumption (2-4 small serves or 1 big + 1 small serve per week) | 61 (16.3%) | 313 (83.7%) |  |
|  | High consumption (all amounts higher than moderate) | 74 (14.6%) | 432 (85.4%) |  |
| Probable serious mental illness (K6) | No | 618 (11.5%) | 4774 (88.5%) | 0.568 |
|  | Yes | 108 (12.2%) | 778 (87.8%) |  |

**Supplementary Table 3. Negative binomial generalised linear models with sociodemographic factors and YFAS symptoms**

|  | Est (95%CI)  Symptom difference (out of a possible 7 symptoms) | p-value |
| --- | --- | --- |
| Model 1. Culturally and linguistically diverse (CALD) - Yes | 0.16 (0.08-0.23) | <0.001 |
| Model 2. School geographic remoteness | -0.06 (-0.18-0.07) | 0.37 |
| Model 3. Relative family affluence (ridit adjusted) - Low | 0.08 (0.02-0.15) | 0.04 |
| Model 4. Relative family affluence (ridit adjusted) - High | 0.02 (-0.05-0.09) |  |
| Model 5. Age* | 0.00 (-0.07-0.07) | 0.93 |
| Model 6. Gender identity* | 0.04 (-0.03-0.11) | 0.26 |
| *Due to low numbers in other categories, only participants aged 12 and 13 and only male- and female-identifying students were included in model. | | |

**Supplementary Table 4. Full results of each negative binomial generalised linear model, adjusting for sociodemographic covariates**

| Table 4.1 SMI |  |  |
| --- | --- | --- |
|  | Est (95%CI) | p-value |
| Serious mental illness (ref category no SMI) | **0.65 (0.58-0.72)** | **<0.001** |
| Low relative family affluence (ref middle) | 0.07 (0.00-0.13) | 0.035 |
| High relative family affluence (ref middle) | 0.03 (-0.04-0.10) | 0.360 |
| Culturally and linguistically diverse (ref category non-CALD) | 0.10 (0.03-0.17) | 0.006 |
| Female (ref category male) | 0.00 (-0.07-0.07) | 0.936 |
| 13-year-old (ref category 12-year-old) | 0.01 (-0.06-0.08) | 0.820 |

| Table 4.2 SSB |  |  |
| --- | --- | --- |
|  |  |  |
|  | Est (95%CI) | p-value |
| SSB: 1 cup or less per week (ref never/rarely drink) | **0.14 (0.07-0.20)** | **<0.001** |
| SSB: 2 to 4 cups per week (ref never/rarely drink) | **0.19 (0.09-0.29)** | |
| SSB: 5 to 6 cups per week (ref never/rarely drink) | **0.30 (0.16-0.45)** | |
| SSB: 7 to 10.4 cups per week (ref never/rarely drink) | **0.41 (0.23-0.60)** | |
| SSB: 10.5 to 13.9 cups per week (ref never/rarely drink) | **0.62 (0.43-0.81)** | |
| SSB: 14+ cups per week (ref never/rarely drink) | **0.83 (0.70-0.97)** | |
| Low relative family affluence (ref middle) | 0.07 (0.01-0.13) | 0.028 |
| High relative family affluence (ref middle) | 0.03 (-0.04-0.09) | 0.430 |
| Culturally and linguistically diverse (ref category non-CALD) | 0.14 (0.07-0.21) | <0.001 |
| Female (ref category male) | 0.11 (0.04-0.19) | 0.002 |
| 13-year-old (ref category 12-year-old) | 0.00 (-0.08-0.07) | 0.898 |

| Table 4.3 Energy drinks |  |  |
| --- | --- | --- |
|  |  |  |
|  | Est (95%CI) | p-value |
| Energy drink: Low consumption - 1 small or big serve or less per week (ref category no consumption) | **0.07 (-0.02-0.17)** | **<0.001** |
| Energy drink: Moderate consumption - 2-4 small serves or 1 big + 1 small serve per week (ref category no consumption) | **0.34 (0.24-0.43)** | |
| Energy drink: High consumption - all amounts higher than moderate (ref category no consumption) | **0.50 (0.41-0.59)** | |
| Low relative family affluence (ref middle) | 0.08 (0.01-0.14) | 0.019 |
| High relative family affluence (ref middle) | 0.02 (-0.05-0.09) | 0.622 |
| Culturally and linguistically diverse (ref category non-CALD) | 0.12 (0.05-0.19) | 0.001 |
| Female (ref category male) | 0.10 (0.03-0.17) | 0.006 |
| 13-year-old (ref category 12-year-old) | -0.01 (-0.08-0.06) | 0.853 |

| Table 4.4 Alcohol |  |  |
| --- | --- | --- |
|  | Est (95%CI) | p-value |
| Consumed at least one standard alcoholic drink in past 6 months  (ref category: no std drink in past 6 mths) | **0.46 (0.30-0.62)** | **<0.001** |
| Low relative family affluence (ref middle) | 0.08 (0.01-0.15) | 0.017 |
| High relative family affluence (ref middle) | 0.03 (-0.04-0.10) | 0.443 |
| Culturally and linguistically diverse (ref category non-CALD) | 0.14 (0.07-0.21) | <0.001 |
| Female (ref category male) | 0.06 (-0.01-0.13) | 0.089 |
| 13-year-old (ref category 12-year-old) | -0.01 (-0.08-0.06) | 0.834 |

| Table 4.5 Tobacco |  |  |
| --- | --- | --- |
|  | Est (95%CI) | p-value |
| Smoked a cigarette in the past 6 months  (ref category: no cigarette use in past 6 months) | **0.51 (0.26-0.76)** | **<0.001** |
| Low relative family affluence (ref middle) | 0.08 (0.01-0.15) | 0.024 |
| High relative family affluence (ref middle) | 0.03 (-0.04-0.10) | 0.427 |
| Culturally and linguistically diverse (ref category non-CALD) | 0.14 (0.06-0.21) | <0.001 |
| Female (ref category male) | 0.06 (-0.01-0.13) | 0.100 |
| 13-year-old (ref category 12-year-old) | 0.00 (-0.08-0.07) | 0.925 |
